# Nuclear Factor (NF) κB polymorphism is associated with heart function in patients with heart failure

**DOI:** 10.1186/1471-2350-11-89

**Published:** 2010-06-09

**Authors:** Diogo GB Santos, Marina F Resende, José G Mill, Alfredo J Mansur, José E Krieger, Alexandre C Pereira

**Affiliations:** 1 Laboratory of Genetics and Molecular Cardiology, Heart Institute (InCor), Sao Paulo University Medical School, Sao Paulo, Brazil; 2 Department of Physiology, Federal University of Espírito Santo, Vitória, Brazil; 3 Cardiology Department, Clinical Division, Heart Institute (Incor), Sao Paulo University Medical School, Sao Paulo, Brazil

## Abstract

**Background:**

Cardiac remodeling is generally an adverse sign and is associated with heart failure (HF) progression. NFkB, an important transcription factor involved in many cell survival pathways, has been implicated in the remodeling process, but its role in the heart is still controversial. Recently, a promoter polymorphism associated with a lesser activation of the *NFKB1 *gene was also associated with Dilated Cardiomyopathy. The purpose of this study was to evaluate the association of this polymorphism with clinical and functional characteristics of heart failure patients of different etiologies.

**Methods:**

A total of 493 patients with HF and 916 individuals from a cohort of individuals from the general population were investigated. The *NFKB1 *-94 insertion/deletion ATTG polymorphism was genotyped by High Resolution Melt discrimination. Allele and genotype frequencies were compared between groups. In addition, frequencies or mean values of different phenotypes associated with cardiovascular disease were compared between genotype groups. Finally, patients were prospectively followed-up for death incidence and genotypes for the polymorphism were compared regarding disease onset and mortality incidence in HF patients.

**Results:**

We did not find differences in genotype and allelic frequencies between cases and controls. Interestingly, we found an association between the ATTG_1_/ATTG_1 _genotype with right ventricle diameter (*P *= 0.001), left ventricle diastolic diameter (P = 0.04), and ejection fraction (EF) (P = 0.016), being the genotype ATTG_1_/ATTG_1 _more frequent in patients with EF lower than 50% (*P *= 0.01). Finally, we observed a significantly earlier disease onset in ATTG1/ATTG_1 _carriers.

**Conclusion:**

There is no genotype or allelic association between the studied polymorphism and the occurrence of HF in the tested population. However, our data suggest that a diminished activation of *NFKB1*, previously associated with the ATTG_1_/ATTG_1 _genotype, may act modulating on the onset of disease and, once the individual has HF, the genotype may modulate disease severity by increasing cardiac remodeling and function deterioration.

## Background

Cardiac remodeling is defined as alterations in left ventricular (LV) chamber mass, geometry and function, and is associated with heart failure (HF) progression[1]. Remodeling leads to LV dilatation and impaired systolic function and transcends the anatomical changes in ventricular geometry, leading to ultra-structural cellular modifications in cardiomyocytes[2,3]. Nuclear factor (NF) κB is a central integrator of stress response and cell survival pathways[4]. Its activation is observed in the myocardial tissue from HF patients of various etiologies[5] suggesting that inflammatory pathways are operant in the development of HF[6].

The Rel/NFκB transcription factor is from a family of evolutionary conserved proteins that are all related through the highly conserved Rel homology binding domain. There are five members of the NFκB family in mammals: p50/p105, p65/RelA, cRel, RelB and p52/p100[7]. In general, transcription activation is dependent on subunit's dimerization. In several tissues, including the heart, the major form of NFκB is the heterodimer p50/p65[8]. The p50 subunit (50 kDa) is encoded in humans by the gene *NFKB1 *and corresponds to the N-terminus of the cytoplasmic protein p105 (105 kDa)[9]. NFκB primarily resides inactive in the cytoplasm by association with IκB. Its activation can be triggered by a variety of stimuli that ultimately lead to phosphorylation, ubiquitination and degradation of IκB, releasing NFκB dimers to nuclear translocation, where NFκB dependent transcription of a large and diverse array of target genes can be initiated and various physiological and pathological processes modulated [10].

*NFKB1 *transcription activation is associated with the development of several diseases such as asthma, inflammatory arthritis, septic shock, lung fibrosis, cancer, AIDS, inflammatory bowel disease, atherosclerosis, diabetes and stroke[10,11], mainly acting by transcription activation of inflammatory processes[12], but the role of its activation in the heart remains controversial. First, NFκB was believed to act, in general, worsening cardiac remodeling or dysfunction mainly through activation of a pro-inflammatory pathway enhancing HF[13-17]. However, recently, several studies have found evidence pointing to a new role for NFκB in the HF scenario. In these studies, NFκB is thought to play a protective role by acting in an anti-inflammatory pathway, participating in matrix remodeling and attenuating oxidative stress thus mitigating the HF progression [18-21].

Recently, two different in vitro studies observed that an insertion (ATTG_2_) of an ATTG sequence at position -94 of the promoter region of the *NFKB1 *gene increases transcriptional activity compared to the allele with the deletion (ATTG_1_)[22,23]. In humans, the production of the p105 and p50 proteins has been shown to be greater among individuals with the ATTG_2_/ATTG_2 _genotype [23]. The purpose of the present work was to study the association of this functional polymorphism with different phenotypes related to HF through an evaluation of the clinical characteristics of 493 individuals with HF of different etiologies.

## Methods

### Study population

Five-hundred and three (503) patients with heart failure in functional class III or IV of the New York Heart Association were studied. The patients were included as part of a secondary-cohort of HF individuals at a tertiary cardiology care center in Sao Paulo, Brazil (Heart Institute of the Sao Paulo University Medical School). Ascertainment period was from August, 2002 to March, 2004.

The diagnosis of heart failure was made according to previously published criteria [24,25]. The classification of the etiologies of heart failure followed previous recommendations[25]. As such, the diagnosis of chronic heart failure was made through both clinical and imaging procedures when necessary. Ischemic cardiomyopathy diagnosis was made when a clear history of previous myocardial infarction and no other probable cause of heart dysfunction was present or, alternatively, through coronary angiography. All patients with the final diagnosis of idiopathic dilated cardiomyopathy were studied through coronary angiography to exclude the possible diagnosis of ischemic cardiomyopathy. In the present analysis we have used data from 493 participants.

### Follow-up

Beginning of follow-up was defined as enrollment in the protocol. Follow-up was assessed in the last outpatient medical visit or by telephone contact. In addition, the mortality database of São Paulo City Authority was also scrutinized to discover patient deaths (ProAim--Programa de Aprimoramento de Informações de Mortalidade do Municipio de São Paulo). Last follow-up was evaluated in April, 2009. Primary end-point studied was overall mortality.

### Control population - General population of Vitória/ES, Brazil

A cross-sectional study of risk factors for cardiovascular diseases was performed in the urban population of Vitoria, Brazil, using the WHO-MONICA project guidelines [26]. The study design was based on cross-sectional research methodology and was developed by means of surveying and analyzing socioeconomic and health data in a probabilistic sample of residents aged 25 to 64 years from the municipality of Vitoria, ES, Brazil. The population was randomized and the sample was socioeconomically, geographically and demographically representative of the residents of this municipality. A selection of 2,268 residential homes located in Vitoria was made and these were visited. The project received approval from the Ethics Committee of the Biomedical Center of Universidade Federal do Espírito Santo (UFES). The selected individuals were asked to attend the Cardiovascular Investigation Clinic of the University Hospital for tests to be performed on the following day. Of the total sample, 1,577 individuals attended. Participants were submitted to physical examination. Major cardiovascular risk factors such as smoking habits, alcohol intake, sedentarism, diabetes and hypertension were inquired. Blood glucose, total cholesterol, lipoprotein fractions, and triglycerides were assayed by standard techniques in a 12-hour fasting blood sample. In the present analysis we have used data from 916 participants.

### Left ventricular function assessment

Left ventricular ejection fraction was determined by M-mode echocardiography. In some patients two-dimensional method was employed, using Simpson. The evaluation of the left ventricular function was performed by the echocardiography staff in a blinded way in relation to the genotypes and was conducted according to previously published recommendations[27].

### Genotype determination

Extraction of genomic DNA was performed from leukocytes separated from whole blood using a standard method[28]. DNA samples were further diluted with PCR grade water to a concentration of 10 ng/μL. The -94 insertion/deletion polymorphism was detected by a High Resolution Melt technique. The primer sequences were F 5'-CATGACTCTATCAGCGGCACT-3' and R 5'-GGCTCTGGCTTCCTAGCAG-3'. Primers were designed using accession number [GenBank: AF213884.2] including a region that does not possess other polymorphisms than the rs28362491 and to be annealed at 60°C using the Primer3 software. The final optimal reaction conditions were empirically determined. The reaction mixture used BioTaq DNA Polymerase (BioQuimica, Brazil) and consisted of 10 ng of genomic DNA, 1× Assay buffer, 2 mM MgCl_2_, 200 nM of each primer, 200 μM of dNTPs, 1,5 μM of SYTO9 (Invitrogen, Carlsbad, USA), 0.5 U of BioTaq DNA Polymerase and PCR grade water in a volume of 10 μL. PCR cycling and HRM analysis was performed on the Rotor-Gene™ 6000 equipment (Qiagen, Courtaboeuf, France). The PCR cycling conditions were as follows: one cycle of 94°C for 5 minutes; 35 cycles of 94°C for 20 seconds, 60° for 20 seconds, 72°C for 20 seconds; and one cycle of 72°C for 5 minutes and an HRM step from 72 to 94°C rising at 0.1°C per second. Curve genotypes were confirmed by an electrophoresis run on a 4% agarose gel (UltraPure™ - Agarose 1000, Invitrogen, Carlsbad, USA) stained with ethidium bromide (1 μg/ml). Ten percent of the samples were randomly chosen and re-genotyped and agreement was of 100%.

### Statistical analysis

Data are presented as means ± standard deviation (SD) for continuous variables and as frequencies for categorical variables. Differences in baseline characteristics among groups were analyzed using ANOVA for continuous variables and the Chi-square test for categorical variables. Hardy-Weinberg equilibrium was evaluated by a Chi-square test.

The probability of survival and disease onset was evaluated by the Kaplan Meier method relative to clinical variables and relative to genotypes of studied polymorphism. Comparisons were made with the log-rank test. Death (overall) was the primary end-point.

Statistical analyses were performed with the SPSS software 13.0. A p value < 0.05 was considered significant.

### Ethics

The investigation conforms to the principles outlined in the Declaration of Helsinki and the study protocol was approved by the Ethics Committee for Medical Research on Human Beings of the *Hospital das Clínicas *from University of São Paulo Medical School. Signed informed consent was obtained from all participants from both samples.

## Results

### Genotyping the rs28362491 polymorphism

Genotype status was obtained for 493 and 916 individuals of the HF and Control samples, respectively. All genotype frequencies were consistent with those predicted by the Hardy-Weinberg equilibrium. Table 1 shows allelic and genotype frequencies among patients and controls where ATTG_1 _and ATTG_2 _represent the allele with the deletion and insertion, respectively. Neither the allelic, nor the genotype frequencies revealed any difference between patients and controls.

**Table 1:** Allelic and genotype frequencies of *NFKB1 *-94 insertion/deletion among pacients and control.

	Patients N = 493	Controls N = 916	p value
Genotype Frequencies			
ATTG_2_ATTG_2_	0.345	0.345	
ATTG_2_ATTG_1_	0.507	0.509	0.99
ATTG_1_ATTG_1_	0.148	0.146	
Allelic Frequencies			
ATTG_2_	0.59	0.575	0.93
ATTG_1_	0.41	0.425	

### Clinical and demographic evaluation

We tested both patient and control samples for association with clinical and demographic characteristics (Tables 2 and 3, respectively). The only significant association was observed between diabetes and the ATTG1 allele in the HF population.

**Table 2:** Demographic and clinical characteristics of the HF population according to -94 insertion/deletion genotype.

	ATTG_2_/ATTG_2_	ATTG_1_/ATTG_2_	ATTG_1_/ATTG_1_	p value
Patients	170	250	73	-
Age, years	59 ± 15.2	58.2 ± 14	54.7 ± 13.7	0.09
Gender				
Male n (%)	95 (56)	137 (62)	53 (59)	0.45
Ethnicity n (%)				
Blacks	12 (23.5)	29 (57.0)	10 (19.5)	
Mulattos	26 (38.8)	26 (38.8)	15 (22.4)	0.08
Whites	129 (35.5)	185 (51.0)	48 (13.5)	
Biochemical				
Serum sodium (mmol/L)	59.61 ± 1.9	59.45 ± 2.0	59.48 ± 1.7	0.72
Hemoglobin (g/dL)	13.03 ± 2.3	13 ± 2.1	13.48 ± 2	0.19
Total Cholesterol (mg/dL)	194.95 ± 50.4	185.53 ± 50.9	195.85 ± 56.6	0.17
Triglycerides (mg/dL)	123.55 ± 67.2	124.5 ± 66.4	119.15 ± 7	0.87
HDL (mg/dL)	47.22 ± 14.7	43.77 ± 15.8	45.61 ± 17.1	0.13
LDL (mg/dL)	122.74 ± 42.9	117 ± 39.5	123.7 ± 48.5	0.41
Creatinin (mg/dL)	1.36 ± 0.72	1.35 ± 0.75	1.33 ± 0.78	0.95
Diabetes n (%)				
Yes	33 (35.5)	54 (54)	6 (6.5)	0.035
No	125 (34.4)	177 (49)	61 (16.6)	
BMI (kg/m^2^)	25.36 ± 5.9	26.1 ± 5.2	24.2 ± 4.93	0.07
Heart Rate (pm)	79.3 ± 13.8	79.5 ± 12.2	80.2 ± 14.6	0.9
Diastolic Blood Pressure (mmHg)	73.9 ± 16.4	76.4 ± 18.4	78.5 ± 20.9	0.19
Systolic Blood Pressure (mmHg)	120.4 ± 30	121.5 ± 30.6	125.6 ± 31.5	0.58

**Table 3:** Demographic and clinical characteristics of the Control population according to -94 insertion/deletion genotype.

	ATTG_2_/ATTG_2_	ATTG_1_/ATTG_2_	ATTG_1_/ATTG_1_	p value
Number	316	466	134	-
Age, years	44.36 ± 10.46	45.01 ± 10.6	43.95 ± 10.54	0.5
Gender				
Male n (%)	136 (43)	171 (36.7)	58 (43.3)	0.14
Ethnicity n (%)				
Blacks	18 (31)	28 (48.3)	12 (20.7)	
Mulattos	150 (33.1)	237 (52.3)	66 (14.6)	0.35
Whites	122 (38.6)	151 (47.8)	43 (13.6)	
Biochemical				
Hemoglobin (mg/dL)	13.83 ± 1.47	13.61 ± 1.41	13.74 ± 1.51	0.12
Total Cholesterol (mg/dL)	215.43 ± 40.73	218.52 ± 43.87	214.16 ± 49.9	0.47
Triglycerides (mg/dL)	124.07 ± 82.3	130.6 ± 92.12	139.24 ± 91.21	0.23
HDL (mg/dL)	45.46 ± 11.91	46.46 ± 12.06	44.73 ± 11.42	0.25
LDL (mg/dL)	145.59 ± 37.76	146.77 ± 39.29	142.32 ± 44.17	0.52
Creatinin (mg/dL)	0.97 ± 0.20	0.96 ± 0.02	0.98 ± 0.17	0.84
Diabetes n (%)				
Yes	22 (29)	45 (58)	10 (13)	0.37
No	294 (35)	420 (50)	123 (15)	
BMI (kg/m^2^)	26.34 ± 5.08	26.55 ± 5.08	26.47 ± 4.9	0.84
Heart Rate (pm)	70.40 ± 12.13	70.81 ± 10.46	70.73 ± 8.94	0.95
Diastolic Blood Pressure (mmHg)	83.27 ± 13.06	82.57 ± 13.45	81.73 ± 12.26	0.51
Systolic Blood Pressure (mmHg)	126 ± 20.22	128.6 ± 22.29	127.77 ± 22.45	0.25

### Functional Evaluation

Patients possessing the ATTG_1_/ATTG_1 _genotype presented higher right and left ventricular diameters demonstrated by higher right ventricle (RV) diameter (*P *= 0.001), left ventricle (LV) diastolic diameter (*P *= 0.04) and a tendency towards higher mean LV systolic diameter (*P *= 0.06). RV and LV remodeling was accompanied by functional deterioration, as can be observed by the lower ejection fraction (EF) in patients with the ATTG_1_/ATTG_1 _genotype (P = 0.016). Cardiac remodeling and functional deterioration were not observed in the control population (Table 4). However, it is interesting to note a non-significant tendency of lower mean EF in carriers of the ATTG_1_/ATTG_1 _genotype in control individuals (p = 0.10).

**Table 4:** Functional data for the HF and Control population according to -94 insertion/deletion genotype.

	**Patients**				**Controls**			
	**ATTG_2_/ATTG_2_**	**ATTG_1_/ATTG_2_**	**ATTG_1_/ATTG_1_**	**p value**	**ATTG_2_/ATTG_2_**	**ATTG_1_/ATTG_2_**	**ATTG_1_/ATTG_1_**	**p value**
	
LAD, mm	46 ± 9.2	46.7 ± 8.8	48.9 ± 8.7	0.09	33.31 ± 3.1	33 ± 2.86	33.83 ± 3.42	0.3
AD, mm	32.8 ± 6.4	32.6 ± 5.2	32.3 ± 6.9	0.88	32 ± 2.9	31.32 ± 2.86	31.58 ± 2.74	0.18
RVD, mm	25 ± 7.73	27.07 ± 8.10	31.07 ± 7.83	0.001	16.55 ± 4.33	17.29 ± 4.77	17.27 ± 4	0.43
Left Ventricle								
IS, mm	9.8 ± 2.5	9.7 ± 2.2	9.70 ± 2.1	0.81	8.98 ± 1.27	8.93 ± 1.15	8.91 ± 1.10	0.9
PWT, mm	9.6 ± 2.1	9.5 ± 1.9	9.5 ± 2	0.95	8.92 ± 1.22	8.75 ± 1.08	8.77 ± 1	0.38
DD, mm	60.8 ± 11.5	61.6 ± 11.4	65 ± 11.3	0.04	48.09 ± 4.84	48.58 ± 4.8	48.76 ± 5	0.6
SD, mm	48. ± 13.7	49.3 ± 14.3	53.5 ± 15.1	0.06	28.7 ± 3.74	29.26 ± 4.10	29.74 ± 4.82	0.24
EF, (%)	47.9 ± 18.5	45.6 ± 19	39.5 ± 18.2	0.016	71.63 ± 4.77	71 ± 5.2	69.70 ± 6.85	0.1
Mass, g	247 ± 104	249 ± 89	267 ± 88	0.36	151 ± 47.87	151 ± 42.7	152 ± 46.9	0.99

LAD - Left Atrium Diameter, AD - Aortic Diameter, RVD - Right Ventricle Diameter, IS - Intraventricular Septum, PWT - Posterior Wall Thickness, DD - Diastolic Diameter, SD - Systolic Diameter, EF - Ejection Fraction

We further analyzed differences in genotype frequencies between patients selected based on an EF above or below 50%. Frequencies in patients with EF > 50% were: 40% ATTG_2_/ATTG_2_, 52% ATTG_1_/ATTG_2 _and 8% ATTG_1_/ATTG_1_, while in patients with EF < 50% they were: 32% ATTG_2_/ATTG_2_, 49% ATTG_1_/ATTG_2 _and 19% ATTG_1_/ATTG_1_. Genotype ATTG_1_/ATTG_1 _was more frequent in patients with EF lower than 50% (*P *= 0.01).

**Figure 1:**
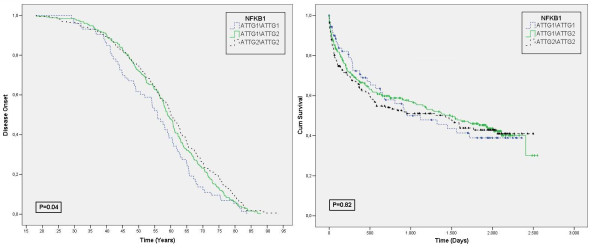
**A Kaplan-Meier curve for Disease Onset (A) and Mortality (B)**. Blue line represents genotype ATTG_1_/ATGG_1_, Green line genotype ATTG_1_/ATTG_2 _and black line genotype ATTG_2_/ATTG_2_. In B, the different forms represent the event death for each genotype.

Finally, we observed that individuals who carry the ATTG_1_/ATTG_1 _genotype presented an earlier disease onset. Figure 1 panel A shows the Kaplan-Meier curves for disease onset in individuals harboring the different *NFKB1 *genotypes. Interesting, those who have the ATTG_1_/ATTG_1 _genotype presented the disease, on average, 7 years sooner than those with the ATTG_2_/ATTG_2 _genotype. No significant difference was observed in Kaplan-Meier curve for mortality (Figure 1, panel B).

## Discussion

The role of NFκB in heart failure has been studied by several authors[6] and it still remains controversial. Many studies applied different methods to model the role of NFkB in the heart failure scenario. These ranged from coronary artery ligation to mouse and rat models that block the *NFKB1 *gene [13-17,19]. Beyond its interaction with other subunits, NFκB can act in the cell by two other forms, the p105 and the homodimer (p50)_2_. The p105 forms complexes with non-NFκB family proteins providing a crosstalk between NFκB and other signaling pathways that regulate different cellular functions[29]. The (p50)_2_, first considered a repressor of transcription, is now believed to also act as a transcription activator. Ultimately, the processing of p105 is in part dependent on the setting of NFκB dimmers in the surroundings, differing in each kind of cell[30]. The complex transcriptional and post-translational modification of the *NFKB1 *gene and product, respectively, might be the reason why the several studies addressing ablation of the *NFKB1 *gene present different conclusions: sometimes observing that NFκB (p50) acts worsening HF by activation of a pro-inflammatory pathway[13-16] whereas in other, protecting the heart by activation of anti-inflammatory pathways, matrix remodeling or attenuation of oxidative stress[19].

In the present study, we evaluated the role of a polymorphism that was previously described to be associated with diminished transcription of *NFKB1 *due to a four base pair deletion in the promoter region of the gene and resulting in the loss of binding of nuclear proteins, thus, leading to a reduced promoter activity.

The rs28362491 polymorphism was first described by Karban et al[22] and associated to an increased risk for ulcerative colitis. This polymorphism has been associated with diverse human diseases but, only recently, Zhou et al. described an association with heart disease[31]. They observed, in 177 patients with dilated cardiomyopathy (DCM), a higher prevalence of the genotypes ATTG_2_/ATTG_2 _+ ATTG_1_/ATTG_2 _in DCM patients, indicating that ATTG_2 _carriers have increased risk of DCM. However, they only observed a non-statistical higher ATTG_2 _allelic frequency in DCM patients (62.7 vs. 57.1). Herein, we used diverse HF etiologies, including DCM, and did not observe neither genotype nor allelic distribution differences between patients and controls (*P *= 0.99 and *P *= 0.96, respectively). This lack of association was also observed when we compared only patients with the DCM etiology (n = 50) with controls (*P *= 0.13 genotype frequencies, *P *= 0.37 allelic frequencies, data not shown), and, despite the low statistical power due to the small number of patients with this specific diagnosis, we observed that the ATTG_2 _allelic frequency in DCM patients is lower than in control individuals (50% vs. 57.5%), indicating an opposite direction for the putative association. These data suggest that the presence of the ATTG_2 _allele itself does not represent a risk factor for DCM or HF in general in our population.

Even considering the problems of the *NFKB1 *ablation models, they are useful to point towards interesting aspects of NFkB physiology in the heart. Recently, Timmers et al[19] reported enhanced cardiac remodeling and dysfunction in NFκB p50 knockout mice after myocardial infarction. Knockout mice presented reduced ejection fraction and higher LV diastolic and systolic volume, indicating cardiac remodeling and deteriorated function. In our analyses we observed a significant difference in EF, LV diastolic and RV diameter only in HF patients (*P *= 0.016, P = 0.04 and P = 0.001, respectively). Signs of impaired heart function were all associated with the ATTG_1_/ATTG_1 _genotype. Additionally, we observed a tendency in LV systolic diameter, LA diameter and mass to be higher in the ATTG_1_/ATTG_1 _genotype. These data corroborate Timmers', and is also supported by another recent study where a model with attenuated activation of NFκB presented similar cardiac modifications [18]. Curiously, although a low EF is known to be the strongest predictor of mortality in HF, we did not observe an association of the ATTG_1 _allele with survival in this patient cohort (P = 0.8) (Figure 1, panel B). Interestingly, the same was observed in p50 KO mice and in mice with attenuated expression of the *NFKB1 *gene[18,20]. Nevertheless, we demonstrated that carriers of the ATTG_1_/ATTG_1 _genotype exhibit a significant anticipated disease onset (of nearly 7 years). Taken together, these data suggest that the presence of ATTG_1_/ATTG_1 _genotype is involved with an enhanced cardiac remodeling process and impaired heart function. The implications of this association to long term prognosis remain to be explored.

The main limitation herein is the lack of replication of the significant associations in another equivalent, independent cohort. This, beyond the confirmation of data, would also give other important information regarding survival and may shed light into why we were not able to observe the expected higher mortality in patients with the ATTG_1_/ATTG_1 _genotype.

## Conclusion

There is no genotype or allelic association between the studied polymorphism and the development of HF. However, our data suggest that a diminished activation of *NFKB1*, previously associated with the ATTG_1_/ATTG_1 _genotype, may act modulating the onset of disease and, once the individual has HF, the genotype may modulate disease severity by increasing cardiac remodeling and function deterioration.

## Competing interests

The authors declare that they have no competing interests.

## Authors' contributions

DGBS carried out the molecular genetic studies, statistical analysis and drafted the manuscript. ACP participated in the design of the study, statistical analysis and coordinated experiments and manuscript preparation. JEK participated in the design of the study. JGM and AJM were responsible for patient selection and characterization. All authors read and approved the final manuscript.

## Pre-publication history

The pre-publication history for this paper can be accessed here:

http://www.biomedcentral.com/1471-2350/11/89/prepub
